# On the diversity and richness of understory bryophytes at Nectandra Cloud Forest Reserve, Costa Rica

**DOI:** 10.3897/BDJ.5.e11778

**Published:** 2017-03-24

**Authors:** Daniel H Norris, Ekaphan Kraichak, Allen C Risk, Diane Lucas, Dorothy J Allard, Frida Rosengren, Theresa A Clark, Nicole Fenton, Michael Tessler, Nonkululo Phephu, Evelyne T Lennette

**Affiliations:** 1 Emeritus, University Herbarium, University of California, Berkeley, California, United States of America; 2 Kasetsart University, Bangkok, Thailand; 3 Department of Biology and Chemistry, Morehead State University, Morehead, KY, United States of America; 4 Kent University Herbarium, Wellington, OH, United States of America; 5 University of Vermont, Bakersfield, VT, United States of America; 6 Lund University, Lund, Sweden; 7 School of Life Sciences, University of Nevada, Las Vegas, NV, United States of America; 8 Institut de recherche sur les forêts (IRF), Université du Québec en Abitibi-Témiscamingue, Québec, Canada; 9 Richard Gilder Graduate School, American Museum of Natural History, New York, NY, United States of America; 10 Tshwane University of Technology, Pretoria, South Africa; 11 Nectandra Institute, San Ramon, Alajuela, Costa Rica

**Keywords:** Tropical bryophytes, bryophyte surveys, mosses, liverworts, Nectandra Cloud Forest Reserve, premontane cloud forest, Costa Rica

## Abstract

**Background:**

A survey of the understory bryophytes in the Nectandra Cloud Forest Preserve yielded 1083 specimens distributed among 55 families, represented by 74 genera of mosses, 75 genera of liverworts and 3 of hornworts. We studied and analyzed the bryophytic distribution on six types of substrates: 1) corticolous, 2) epiphyllous, 3) saxicolous, 4) terricolous, 5) aquatic and 6) lignicolous. The richness and composition of bryophyte genera are compared to those of other previous bryophyte surveys from 4 other sites with different oceanic exposures, climatic and geographic conditions in Costa Rica.

**New information:**

This is a report of the first extensive general survey of bryophytes at the Nectandra Reserve, a premontane cloud forest located on the Atlantic slope of Costa Rica, an area much less studied compared to the Monteverde cloud forest on the Pacific slope.

## Introduction

Costa Rica’s climate and weather are determined by the Atlantic and Pacific oceanic influences driven across a very narrow landmass with a backbone of volcanic ranges. Orographic uplifting of the dominant northeasterly Atlantic wet trade winds, which directly impinge against the Cordillera Tilarán, result in intense precipitation on the mid-to-upper Atlantic slope (1000-3000m) in the form of unremitting rain and wind-driven cloud during most of the year, peaking in December-February. In comparison, the west-facing Pacific slopes are steeper, on the leeward side of the trade winds, hence are drier with fractured regions of cloud coverage (Oliveira et al. 2014). However, the most important features that characterize premontane cloud forests, such as the current study site, are the predictable and prolonged daily cycles of cloud immersion (Nadkarni and Wheelwright 2000, Nair et al. 2008, Lawton et al. 2010, Bruijnzeel et al. 2010), the intensity of wind-driven rain and cool temperature — conditions that affect plant ecophysiology, e.g. evapotranspiration foliar water uptake leaf area, and tree height (Nadkarni and Wheelwright 2000, Clark et al. 1998, Clark et al. 2005, Goldsmith et al. 2013). The highly variable nature of clouds, in addition, generates unique but wide ranging microclimates and ecology, which favor high biodiversity density, epiphytic and canopy stratification (Clark et al. 2000, Clark et al. 2014).

**Figure 1.**
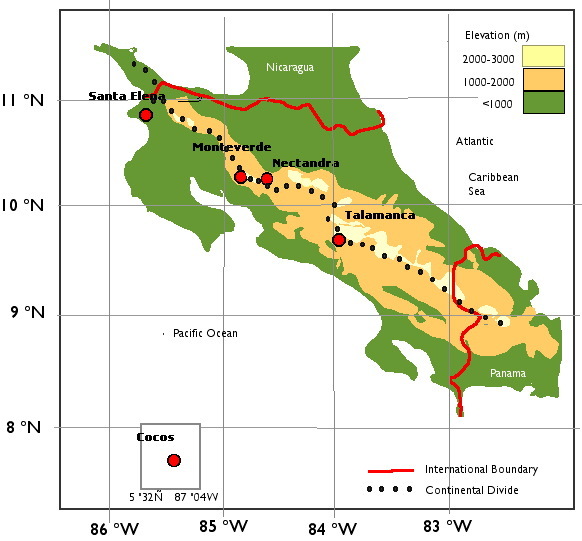
Published general bryophyte surveys in Costa Rica

The richness of tropical bryophytes in cloud forests is overwhelming. Bryophytes are found mostly in complex tangles, in long, heavy aerial strands, or in thick mats on all surfaces starting from the ground all the way to the top of the forest canopy, with an abundance of epiphyllous bryophytes in between. As a result, bryophyte ecology is not well studied and its enormous diversity is only beginning to be appreciated. Increasing number of recent reports highlighted the multiple and complex ecologic roles the bryophytes play in the tropical forest. Through poikilohydry, bryophytes resist desiccation and trap water after rehydration. They retain, fix and cycle free atmospheric inorganic nitrogen, carbon and ions (Turestky 2003, Clark et al. 2005, Matzek and Vitousek 2003). They act as nutrients, ions and gas exchanges (Coxson 1991), they trap soil particles, stabilize soil to provide complex food and habitats to a host of organisms and microorganisms (Read et al. 2000). Through their adaptive growth habits in a wide range of conditions and substrates, they ultimately influence the ecosystem succession of their environment (Fig. 1).

Current area-based floristic information of Costa Rican bryophytes can be gleaned from published, general surveys at four main localities (Fig. 1) — the Monteverde Cloud Forest Reserve (Gradstein et al. 2001b, Merwin et al. 2001), Los Robles (Oak Forest) in the Cordillera Talamanca (Holz et al. 2002), dry forest in the Santa Elena Peninsula (Dauphin and Grayum 2005) and the Cocos Island (Dauphin 1999). Based on the Holdridge Life Zones (Holdridge 1967), these four study areas are classified respectively as tropical montane cloud forest (Monteverde, 1500 m elevation), the upper montane tropical oak forest (Los Robles Reserve, Rio Sevegre watershed in the Cordillera Talamanca, 2200-2500 m elev), tropical dry forest/lower montane forest (Santa Elena Peninsula), and insular volcanic island forest (Cocos Island, 0-600m, 500km from the Costa Rica Pacific coast). The latter three localities all have Pacific exposure, whereas the Monteverde Cloud Forest Reserve straddles the Continental Divide of the Tílarán Volcanic range, although most of its area is on the Pacific slope.

Detailed botanic studies at the Monteverde Cloud Forest Reserve showed that the vegetation richness on the Pacific slope is slightly over half of that on the wetter Atlantic slope, where plant diversity increases with the moisture gradient from mid-to-high (700-1500 m) elevation (Nadkarni and Wheelwright 2000). Interestingly, the principal work on the Monteverde bryophytes were carried out in study plots at 1500m near the Continental Divide, but on the Pacific slope. We wanted to see if the different Atlantic vs Pacific vegetation richness also applies specifically to the non-vascular bryophytes.

Nectandra Cloud Forest Reserve (henceforth Nectandra) is a private reserve dedicated to cloud forest conservation on the Atlantic slope of the Cordillera de Tílarán, at 1100 -1200m elevation. It is located 40km southeast of the Monteverde Cloud Forest Reserve. Nectandra’s Atlantic exposure, lower elevation, and proximity to Monteverde Cloud forest Reserve presented us with the opportunity to compare the bryoflora of the lower but wetter Atlantic slopes to that of the more studied Monteverde Cloud Forest Reserve. At Nectandra, the higher average precipitation, more moderate temperature and lower elevation are all favorable conditions for higher bryodiversity compared to the Monteverde Reserve. Our survey will hopefully provide a useful comparison of bryoflora from five databases for future research on the effects of climate change on cloud forest.

## Materials and Methods

### Study Area

The 158 ha Nectandra Reserve (10°11'N, 84°31'W, elevation 1100 to 1200m) has an east-west axis along the 2 km arm of the L-shaped property. The vegetation is primary humid premontane forest (Holdridge Life Zone System 1967), with 98% forest canopy that occupies three-fourths of the property. The remaining one-fourth is a naturally regenerating forest (post 1980) on former coffee and *Dracaena* plantations. Two permanent creeks and four intermittently wet drainage streams cross the property to drain into the Balsa River. A network of narrow footpaths allows access to much of the mature forest interior and also its entire perimeter. A second smaller network of surfaced trails (gravel, paving stones and asphalt) surrounds the visitor facilities on the east end of the reserve.

Nectandra is subclassified as tall-statured montane cloud forest based on biologic and hydrometeorologic variables (Bruijnzeel et al. 2010). It has an average rainfall 3000-3500mm y^-1^ and an estimated 80% fog saturated days. During the wet season (May–Oct), cloudy, overcast mornings with torrential afternoon rain are typical. During the wetter season (Nov- Feb) continuous rain with strong, wind-driven, heavy mist may last several weeks to months. Intermittent sunny to overcast days are mainly seen during the dry season (Mar-Apr). Volcanic basalt rocks, layered above a base of lava and volcanic agglomerates, cover much of the reserve. This thick matrix of volcanic ash form the clayey, vertical walls of many small canyons and eroded gullies.

### Vascular Plant Communities

The vascular plant diversity was determined from 30 permanent survey plots (10m x 20m) randomly distributed over the entire property (unpublished data). All the vascular plants in each plot were identified, tallied and tagged for monitoring. Of the total of 918 trees, at least 128 distinct species were identified. The most dominant plants in order of decreasing abundance included tree ferns *Alsophila firma, Cyathea schiediana, Alsophila imrayana)*, non-ferns *Guettarda poasana, Ocotea tonduzii, Conostegia oerstediana, Elaegia auriculata*, each with species density averaging at least two individuals per plot. The 26 genera with the largest trees include *Guarea* (range 47–104 cm in diameter), *Dussia, Hedyosmum* (44-100 cm), *Ficus* (47-80 cm), *Ocotea* (42-76 cm) , *Guatteria* (60-75 cm), *Paquira* (40-73 cm), and *Billia* (53 – 76 cm) .

### Sampling Method

Between 2007 and 2009, D.N. made two separate surveys of mainly understory bryophytes at Nectandra. A third effort (2010) was made in conjunction with a tropical bryophyte course, taught by D.N and E.K. It was attended by eleven participants in the Bryophyte Study Group from seven countries, eight of whom contributed to the collection.

Haphazard floristic sampling of mosses, liverworts and lichens was carried out within 4m of 10 km of trails (equivalent sampled area of 4 ha) on all microhabitats ≤2m in height, including soil, soil banks, streams, rocks, tree trunks, branches, twigs, living leaves. The bryophyte growth on all surfaces is typically dense and entangled. One to five gram-size clumps or mats of candidate specimens were removed from the hard or pendant surfaces (bark/trunk, rock/asphalt) and placed in pre-numbered packets for evaluation and identification. The morphology of bryophyte species were examined in the laboratory with Zeiss dissecting and compound microscopes. With mixed clumps, the species of interest was teased away from the main clump and placed in an individual micro-packet for identification. Multiple micro-packets containing different, segregated, individual species of interest were prepared for each clump/leaf collected. Each outer packet hence contained the remainder of the clump and the associated micro-packets of the voucher specimens for the herbarium. The micro-packets in each main packet were differentiated by letters of the alphabet. The collection (see Supplementary material list A) was deposited at the Jepson Herbarium at the University of California at University of California, Berkeley. At the time of this report, 75% of the collection have been accessioned and the sample information are accessible online through www.ucjepson. Only bryophyte specimens with sufficient information and identification, completed to the level of genera were included in this report. To evaluate the completeness of the collection, we calculated the projected number of species, using the species accumulation curve (the function “specaccum”) and the species pool richness estimator (the function “specpool”) in the R-package “vegan”, from the accessions that were identified to the species level (Oksanen et al. 2016).

The identification of the specimens followed the key and description provided in Gradstein et al. 2001a and relevant monographs and literature therein. The nomenclatural information was verified with the TROPICOS database (www.tropicos.org).

## Results

### General Characteristics of Samples

There were a total of 1083 specimens examined at the genus level (Suppl. material 1). We identified 28 moss families (74 genera, 394 specimens), 24 liverworts (75 genera, 671 specimens) and 3 hornworts (3 genera, 18 specimens) families.

**Figure 2.**
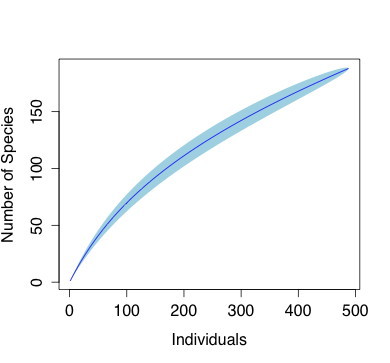
Individual sample-based species accumulation curve from the bryophyte specimens from Nectandra, using the rarefraction method. The light blue band represents 95% confidence interval.

Only a subset of 488 specimens were identified with confidence to the species level (Suppl. material 2), in part due to the lack of complete monographic work in various taxonomic groups. Of these specimens, we found 188 species from 44 families (112 species of mosses, 74 species of liverworts, and 2 species of hornwort. The complete number of species was expected to be between 231-412 species (Fig. 2). However, since the majority of specimens were only identified to the generic level and difference in species identification between sites, we used the genus-level data for the subsequent analysis (Fig. 2)

Fig. 3a shows the composition of the mosses. Eight families accounted for 58% (43/74 genera) of the diversity. These 8 families, listed in the order of decreasing family size, were Dicranaceae, Hookeriaceae, Pilotrichaceae, Hypnaceae, Neckeraceae, Bryaceae, Meteoriaceae, Sematophyllaceae, each composed of 4-8 genera. In contrast, a single liverwort family Lejeuneaceae with 40 genera accounted for 55% (40/75) of the total genera (Fig. 3b). The 3 families of hornworts were represented by a single genus each (data not shown).

### Generalists vs. Specialists

We classified the forest understory into 6 types of microhabitats: saxicolous, epiphyllous, corticolous, terricolous, aquatic rocks (submerged occasionally during heavy rain), and lignicolous. The first five categories were almost exclusively found in the forest understory. The last category, lignicolous, consisted mainly of exposed dry fence posts on the northern boundary adjacent to a treeless deforested ranch. It is the only habitat that receives full sun (Table 1).

**Figure 4.**
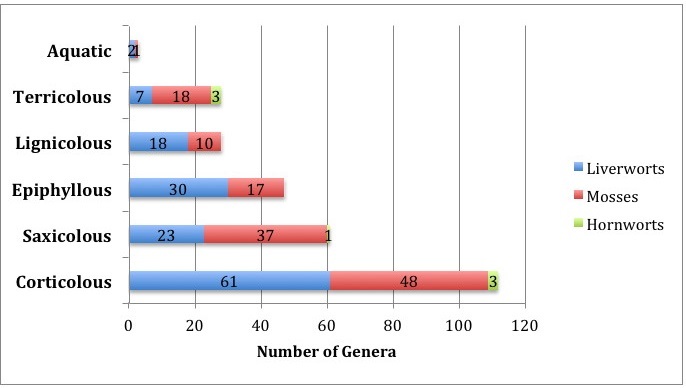
Distribution of bryophytes on six substrates

Table 1 shows the analysis of 152 genera of bryophytes for their distribution in the above 6 habitats. Almost half of the mosses and liverworts were generalists (found on two or more substrate types) and half were specialists (found on a single type of microhabitat). More specifically, 34 of 74 (46%) genera are moss generalists and 35 of 75 (47%) are liverwort generalists. The remaining majority of taxa (40 genera each) of bryophytes are specialists, occupying unique niches in the understory (Fig. 4).

The corticolous bryophytes dominated with 112 genera collected off trunk, branches and twigs (Fig. 4). Together with the 47 epiphyllous and 28 lignicolous taxa, there were 187 genera of epiphytic bryophytes (109 of liverworts, 75 of mosses and 3 hornworts) growing on living or dead trees. In comparison to botanic substrates, only 85 taxa were collected off soil and rocks, with the mosses outnumbering the liverworts almost two to one (55 mosses to 30 liverworts). Hornworts were collected in equal number on soil and rock substrates. Only two genera of liverworts (*Plagiochila* and *Cephaloziopsis*) and one of moss (*Sematophyllum*) were found on boulders occasionally submerged during the heavy rainy season and flooding (Table 2).

### Comparison of Five Locations from Different Life Zones

We made detailed comparisons of the bryological diversity, at the level of genera at Nectandra with 4 other locations previously mentioned in the Introduction. Table 2 shows the locations, geophysical and climatic conditions of all 5 sites and tabulates the total number of bryophyte genera, as well as the numbe of genera in common or different from Nectandra. Compared to Nectandra, Monteverde showed lower number of bryophyte genera (47 mosses and 50 liverworts) but 92% were similar to those at Nectandra. Los Robles, on the other hand, has about the same number of mosses (72 genera) and few liverworts (50 genera), but many more were different (17% of both) from those at Nectandra. Santa Elena 95% of its mosses and 81% liverworts genera in common with Nectandra, with 17- 19% being different. The most intriguing site comparison is Cocos Island, where the 100% of 32 moss genera and 16 of liverworts coincide with those of Nectandra. However, the area of collection on Cocos Island was much smaller (0.08 ha) in comparison to the other study sites. Table 3, Table 4 detail shared and distinct bryophyte genera amongst the five sites.

## Discussion

Our inventory represents the first bryophyte collection at Nectandra and the first general bryophyte survey in a premontane cloud forest on the Atlantic slope of Costa Rica. Geologically, Nectandra and Monteverde are both located on the same volcanic range, the Cordillera Tilarán, within 50km of each other. Floristically, the two reserves share the same principal tree families, Lauraceae and Rubiaceae, as well as a whole host of terrestrial herbs and epiphytes (Nadkarni and Wheelwright 2000). Nectandra encompasses a contiguous, closed canopied, mature forest that covers 98% of the property with only 2% with light gaps of any size. Although the differential east-west incline of the property is only 100m elevation, there are several streams that traverse the property in the south-north direction creating deep ravines and wet stream banks. Except for a few days during March-April, the forest understory and forest floor remain damp throughout the year. Nocturnal low clouds often mist the forest during the driest period of the year. The two most conspicuous features in the preserve are its unbroken, dense epiphytic growth and the heavy, multi-species bryophyte mass on every type of surface.

### Species diversity

From the accessions that we can determine with confidence, a total of 189 species were identified. This current number of species is far from complete, as suggested by increasing slope of the species accumulation curve and the discrepancy from the projected 213-412 species in Fig. 2. The remaining species are possibly among the undetermined specimens, or unexplored habitats, such as canopy or fully submerged habitat.

### Generic density

For practical reasons, we concentrated on the bryophytes accessible without climbing and included living as well as fallen trees. We did not estimate the proportion of specimens associated with freshly fallen trees. Our survey yielded a total of 152 genera of mosses, liverworts and hornworts at Nectandra, with a comparable number of hepatic and moss genera, although the number of genera per family differed markedly for the two groups (Fig. 3). The number of moss genera was evenly distributed across the families, whereas the hepatics showed a single dominant Lejeunaceae with 40 genera. Four liverwort families — Lejeuneaceae, Plagiochilaceae, Lepidoziaceae, Metzgeriaceae — accounted for 50% of the total 1083 specimens, whereas the largest 5 moss families (Sematophyllaceae, Dicranaceae, Calymperaceae, Hookeriaceae, and Polytrichaceae) represented only 17%. The dominance of these families are not surprising, because they are among the most species- and genera-rich families in the tropics (Goffinet and Shaw 2009). Lejeuneaceae, for example, consists of almost 100 genera, all of which are predominantly distributed in the tropics (Gradstein 2013). Two dominant pleurocarpous families (Sematophyllaceae and Hookeriaceae) are still subject to active taxonomic revisions (Hadenas 2012, Pollawatn 2008), and their number of genera could be still changing.

### Mosses compared to hepatics

While there was an almost equal number of genera collected for mosses and liverworts, the ratios of specimens per genus was not — 394/74 for mosses vs. 671/75 for liverworts. This difference can be accounted quantitatively and qualitatively by Lejeuneaceae. Not only were there 40 genera in that single family, 68 % of the genera were generalists, growing on 2 of the 6 substrates tested, hence the large number of total liverwort specimens encountered. At the same time that there was a higher generic diversity in Lejeuneaceae, the number of individuals per genus in this family is lower — 7.5 compared to the average 10.9 for liverworts in the remaining genera and 10.7 for mosses in general. Given that we employed haphazard floristic sampling, the number of specimens should reflect more or less the abundance of bryophytes in the garden. Such a difference in the specimen numbers of mosses and liverworts highlights the tropical nature of bryophyte distribution, in which liverworts tend to be more common than they do in the temperate zone (Pócs 1982).

### Multi-site comparison

Our survey at Nectandra yielded 152 genera compared to 123 for Los Robles, 98 reported for Monteverde, 77 for Cocos and 36 for Santa Elena Peninsula. Given that the methodology and the surveyors differed at each site, it is not possible to make statistically meaningful comparisons. Nevertheless, it is useful to know the outcome of a coarse comparison among the five sites (Tables 2, 3, 4). The bryophyte richness at Los Robles is comparable to that at Nectandra, although they differ by 21 genera, the highest number of non-overlapping taxa among the 5 sites. Santa Elena Peninsula, not unexpectedly, was the least similar to Nectandra, due to the lower elevation and hotter and drier climate. Intriguingly, the bryodiversity on Cocos Island overlaps exactly with that of Nectandra at the genus level. Unfortunately, the survey on Cocos Island was done in a very small area (0.8 ha) and therefore difficult to compare with more certainty. Lastly, our nearest neighbor Monteverde has 90% of the taxa in common with Nectandra, with 9 distinct families. Our results are consistent with a similar but higher diversity at Nectandra than that of Monteverde. It is noteworthy that just in the Lejeuneaceae alone, 40 genera were collected at Nectandra, compared to 27 at Monteverde and 23 at Los Robles. Several families of mosses and liverworts were absent from Nectandra, but found in Monteverde and Los Robles. The absence can probably be attributed to our incomplete sampling from the canopy and other habitats or sampling missed in our survey. *Sphagnum* (Sphagnaceae), for example, only occurs in bogs with poor drainage and acidic water, which is not found in Nectandra. However the number of hornworts taxa at Nectandra was by far the highest among all the sites. Anthocerotaceae was present at Nectandra, Los Robles and on Cocos Island. Dendrocerotaceae was found at Monteverde and Nectandra whereas Notothyladaceae was unique to Nectandra.

## Conclusions

Despite its partial account, our inventory of bryophytes at Nectandra yield the highest number of genera in the area-based studies in Costa Rica to date. The data here will hopefully contribute to a growing database and stimulate further floristic and ecological studies.

## Supplementary Material

Supplementary material 1 List of 1083 bryophyte specimens collected at Nectandra classified to level of genera Data type: Specimen descriptions
Brief description: List of 1083 bryophyte specimens collected at Nectandra Cloud Forest Reserve (2007-2010) with accompanying habitat and collection information. These specimens were identified to genus level
File: oo_115012.xlsx Daniel H Norris

Supplementary material 2 List of Bryophyte Species collected by at Nectandra classified to level of species Data type: Excel file of Specimens list
Brief description: List of 488 of bryophyte specimens collected at Nectandra (2007-2010) with full identification
File: oo_115011.csv Daniel H Norris

## Figures and Tables

**Figure 3a.**
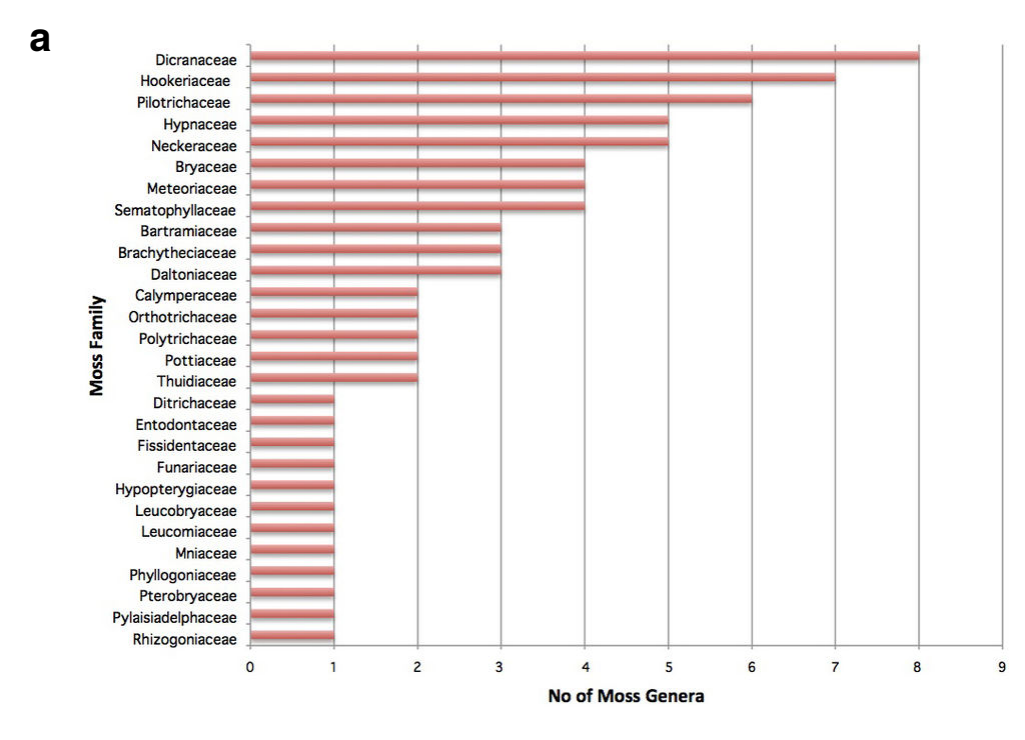


**Figure 3b.**
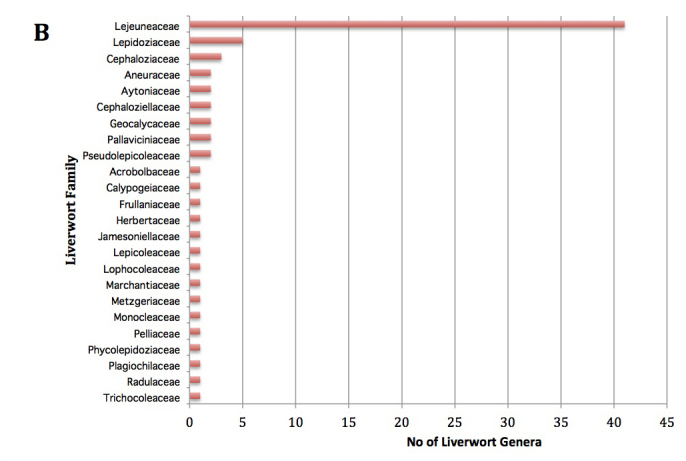


**Table 1.** Distribution of bryophytes among 6 habitats

**No. Substrate**	**Moss**	**No. Specimens**	**Liverwort**	**No. Specimens**	**Hornwort**	**No. Specimens**
Types*	Genera	Collected	Genera	Collected	Genera	Collected
6	0	0	1	97	0	0
5	0	0	1	39	0	0
4	8	30	5	232	0	0
3	9	73	14	140	1	14
2	17	66	14	74	2	4
1	40	225	40	89	0	0
Total	74	394	75	671	3	18

*Substrate Types: Corticolous, epiphyllous, lignicolous, saxicolous, terricolous, and aquatic rocks,

**Table 2.** Study area and comparison of bryophyte richness at five sites

	**Nectandra**	**Monteverde**	**Los Robles**	**Cocos Islands**	**Santa Elena Peninsula**
Citations		Merwin et al. 2001	Holz et al. 2002	Dauphin 1999	Dauphin and Grayum 2005
Exposure	Atlantic	Pacific	Pacific	Pacific	Pacific
Holdridge Life zones	premontane cloud forest	lower montane cloud forest	upper montane cloud forest	lowland and montane cloud forest	dry lowland & wet montane cloud forest
Period of Study	2007-2010	1992-1994	1999-2000	1994	2003
Location in Costa Rica	10°11N, 84°31W	10°18N, 84°48W	9°32N, 83°51W	5°32N, 87°04W	10°57N, 85°45-57W
Elevation in m	1100-1200	1550	2200-2500	0-600	0-700
Survey Method	4m x 10km trails	4 x 1ha plots	8m x 7km trails	8 x 100m2 plots	Not given
Area Surveyed	4 ha	4 ha	6 ha	0.8 ha	Not given
Mean Annual Rainfall (m)	3.5	2.5	2.8	6	1.5
Mean Annual Temp °C	20	18.8	11	25.5	28
Collection height	≤2m	lower canopy	Understorey and	Not Given	Not given
			crown of 7 fallen trees		
Sampling	Haphazard	Random	Random	Quandrants 0.3m x 0.3m	Random
Forest type	Mature	Primary & secondary	Mature Oak	varied	varied
Total Moss Genera	74	47	72	32	20
Shared genera		43 (91%)	60 (83%)	32 (100%)	19 (95%)
Different genera		4 (9 %)	12 (17%)	0 (0%)	1 (5%)
Total Liverwort genera	75	50	50	44	16
Shared genera		46 (92%)	41 (83%)	44 (100%)	13 (81%)
Different genera		4 (8%)	9 (17%)	0 (0%)	3 (19%)
Total hornwort genera	3	1	1	1	0
Shared genera		1	0	1	
Different genera		0	1	0	

**Table 3.** Number of moss genera by family at the five sites. Each dot (•) = one shared genus; Each (o) = genus not at Nectandra.

**Mosses**	**Nectandra**	**Monteverde**	**Los Robles**	**Cocos**	**Santa Elena**
Bartramiaceae	•••	•	•••	•	•
Brachytheciaceae	•••	••	••		
Bryaceae	••••	•••	••••	•	••
Calymperaceae	••	•	•	••	••
Daltoniaceae	•••	••	•••		
Dicranaceae	••••••••	•••••	••••••	••	•
Ditrichaceae	•				
Entodontaceae	•				•
Fissidentaceae	•	•	•	•	•
Funariaceae	•				
Hookeriaceae	•••••••	•	•	••	
Hypnaceae	•••••	••••	••••	•••	•
Hypopterygiaceae	•		•		
Leucobryaceae	•		•	••	•
Leucomiaceae	•		•	•	
Meteoriaceae	••••	••••••	••••••	••	•
Mniaceae	•		•		
Neckeraceae	•••••	•••	••	•	
Orthotrichaceae	••	•••••	•••	•	•
Phyllogoniaceae	•	•	•		
Pilotrichaceae	••••••		••••	•••••	
Polytrichaceae	••		••••		
Pottiaceae	••		•••	•	•••••
Pterobryaceae	•	•	•		•
Pylaisiadelphaceae	•				
Rhizogoniaceae	•	•••	••	•	
Sematophyllaceae	••••	•••	•••	•••••	•
Thuidiaceae	••	•	••	•	
Adelotheciaceae			o		
Amblystegiaceae		o	o		
Cryphaeaceae			o		
Hedwigiaceae			o		
Lembophyllaceae		o	o		
Lepyrodontaceae			o		
Leucodontaceae			o		
Plagiotheciaceae			o		
Prionodontaceae		o	o		
Racopilaceae			o		o
Regmatodontaceae		o			
Sphagnaceae			o		
Splachnaceae			o		

**Table 4.** Number of liverwort genera by family of the five sites. Each dot (•) = one shared genus; Each (o) = genus not at Nectandra.

**Liverworts**	**Nectandra**	**Monteverde**	**Los Robles**	**Cocos**	**Santa Elena**
Acrobolbaceae	•	•	•		
Aneuraceae	••	•	•	••	
Aytoniaceae	••				
Calypogeiaceae	•	•	•	•	
Cephaloziaceae	•••	••	••	•	
Cephaloziellaceae	••			•	•
Frullaniaceae	•	•	•	•	
Geocalycaceae	••		••••	••	
Herbertaceae	•	•	•	•	
Jamesoniellaceae	•	•			
Lejeuneaceae	40	26	23	28	11
Lepicoleaceae	•		•		
Lepidoziaceae	•••••	•••••	••••	•••••	
Lophocoleaceae	•	•			
Marchantiaceae	•		•		
Metzgeriaceae	•	•	•		
Monocleaceae	•		•		
Pallaviciniaceae	••	••	•		
Pelliaceae	•				
Phycolepidoziaceae	•				
Plagiochilaceae	•	•	•	•	•
Pseudolepicoleaceae	••				
Radulaceae	•	•	•	•	
Trichocoleaceae	•	•	•		
Adelanthaceae		o	o		
Balanthiopsaceae			o		
Fossombroniaceae					o
Jubulaceae		o			o
Jungermanniaceae		o	ooo		
Lepicoleaceae			o		
Porellaceae			o		
Ricciaceae					o
Scapaniaceae		o	oo		
